# The impact of infection complications after trauma differs according to trauma severity

**DOI:** 10.1038/s41598-021-93314-5

**Published:** 2021-07-05

**Authors:** Akira Komori, Hiroki Iriyama, Takako Kainoh, Makoto Aoki, Toshio Naito, Toshikazu Abe

**Affiliations:** 1 Department of Emergency and Critical Care Medicine, Tsukuba Memorial Hospital, Tsukuba, Japan; 2 Department of General Medicine, Juntendo University Faculty of Medicine, Tokyo, Japan; 3 Advanced Medical Emergency Department and Critical Care Center, Japan Red Cross Maebashi Hospital, Maebashi, Japan; 4 Department of Emergency Medicine, Gunma University Graduate School of Medicine, Maebashi, Japan; 5 Department of Health Services Research, Faculty of Medicine, University of Tsukuba, Tsukuba, Japan

**Keywords:** Trauma, Infectious diseases

## Abstract

The impact of infection on the prognosis of trauma patients according to severity remains unclear. We assessed the impact of infection complications on in-hospital mortality among patients with trauma according to severity. This retrospective cohort study used a nationwide registry of trauma patients. Patients aged ≥ 18 years with blunt or penetrating trauma who were admitted to intensive care units or general wards between 2004 and 2017 were included. We compared the baseline characteristics and outcomes between patients with and without infection and conducted a multivariable logistic regression analysis to investigate the impact of infection on in-hospital mortality according to trauma severity, which was classified as mild [Injury Severity Score (ISS) < 15], moderate (ISS 15–29), or severe (ISS ≥ 30). Among the 150,948 patients in this study, 10,338 (6.8%) developed infections. Patients with infection had greater in-hospital mortality than patients without infection [1085 (10.5%) vs. 2898 (2.1%), *p* < 0.01]. After adjusting for clinical characteristics, in-hospital mortality differed between trauma patients with and without infection according to trauma severity [17.1% (95% CI 15.2–18.9%) vs. 2.9% (95% CI 2.7–3.1%), *p* < 0.01, in patients with mild trauma; 14.8% (95% CI 13.3–16.3%) vs. 8.4% (95% CI 7.9–8.8%), *p* < 0.01, in patients with moderate trauma; and 13.5% (95% CI 11.2–15.7%) vs. 13.7% (95% CI 12.4–14.9%), *p* = 0.86, in patients with severe trauma]. In conclusion, the effect of infection complications in patients with trauma on in-hospital mortality differs by trauma severity.

## Introduction

Infection after trauma, including sepsis, is the most common complication affecting trauma patients; unfortunately, it has a poor prognosis. However, some infections may be preventable or recognized early because infections are typically acquired during hospitalization after the onset of trauma^1^. Therefore, complication rates and the failure to rescue including infection are considered indicators of the quality of trauma care^2^.

Previous studies have reported that the overall mortality from trauma has gradually decreased in western countries^3^ and in Japan^4^. Additionally, many studies have shown the improvement in outcomes for patients with sepsis following the development subsequent revision of definitions, guidelines, and bundles^5–8^. Conversely, only limited information is available on the development of infection and sepsis in patients with trauma^1^, likely because the definition of sepsis continues to change^9^. Patients with trauma often arrive with organ dysfunction, which adds complexity and inaccuracy to applying the definition of sepsis using organ failure scores such as sequential organ failure assessment scores^10^. It is difficult to determine whether severity scores and organ damage are caused by trauma or the subsequent infection. Furthermore, few studies have assessed the impact of infection and its prognosis among trauma patients. Prognosis among trauma patients complicated with infection may be influenced by trauma severity.

Therefore, we aimed to assess the impact of infection complications on in-hospital mortality among patients with trauma according to trauma severity using a national database in Japan.

## Methods

### Study design and data source

This retrospective cohort study used the Japan Trauma Data Bank (JTDB) database from 2004 and 2017. The JTDB was established in 2003 and is authorized and maintained by the Japanese Association for the Surgery of Trauma and the Japanese Association for Acute Medicine to improve and assure the quality of trauma care in Japan. A total of 272 hospitals, including more than 75% of the certified tertiary emergency medical centers in Japan, contributed to the JTDB in March 2018^11^.

### Data collection

The JTDB includes data related to patient and hospital information such as patient demographics, Abbreviated Injury Scale scores, Injury Severity Score (ISS), prehospital and in-hospital procedures, complications, and treatment and emergency procedures including transfusion within 24 h. The JTDB also records outcome data such as emergency department (ED) mortality, in-hospital mortality, and length of hospital stay. Data collection was performed as a part of routine clinical patient management.

### Study participants

Patients aged ≥ 18 years with blunt or penetrating trauma who were admitted to the intensive care unit or a general ward were enrolled in this study. We excluded patients who died < 7 days after admission, similar to previous reports^12,13^, to exclude the effects of first trauma impact on in-hospital mortality and because infection usually occurred approximately seven days after trauma^14^. We also excluded patients who met the following criteria: missing data on sex and ISS, an Abbreviated Injury Scale score of 6 (i.e., non-survivable injury), inconceivable vital signs in the ED (e.g., systolic blood pressure ≤ 40 mmHg), hospital stay for ≥ 1 year or missing, or missing data on in-hospital death.

### Definitions

Infection and sepsis were clinically diagnosed by a physician in charge. Sepsis was identified a composite variable, “sepsis/multiple organ failure”, in the JTDB database. This definition is similar to the definition of severe sepsis in the Sepsis-2 criteria^15^. We divided trauma severity into three groups based on the ISS to reflect the clinically relevant categories, similar to previous reports^1,16^: ISS < 15 (mild), ISS 15–29 (moderate), and ISS ≥ 30 (severe). Types of infections included pneumonia, urinary tract infection, surgical site infection, myelitis, meningitis, abdominal abscess, cholecystitis, enterocolitis, empyema, and bacteremia. The definition of a complication was in accordance with the JTDB. All emergency procedures were operated as part of the resuscitation or initial management at the ED.

### Statistical analysis

Continuous variables were presented as the median and interquartile range and were compared using the Mann–Whitney U test because none of the variables were normally distributed. Categorical variables were presented as numbers and percentages and compared using the Chi-square test. We compared the baseline characteristics such as age, sex, site of injury, comorbidities, emergency procedures, concomitant complications, and outcomes between the patients with and without infection.

We performed a multivariable logistic regression analysis to investigate the influence of infection on in-hospital mortality for trauma patients. The adjusted variables included age, sex, number of comorbidities, transfusion, emergency procedures, admission disposition, any operations, and concomitant complications; these variables were chosen based on previous reports and clinical relevance^1,16–18^. We assessed the multicollinearity of variables using the variance inflation factor, and the tolerance value was set at less than 2. We then used marginal standardization based on probability determined from the previous analysis to estimate the adjusted in-hospital mortality rate according to trauma severity. The results were reported as adjusted in-hospital mortality rates with 95% confidence intervals (CIs). In a subgroup analysis, we evaluated the impact of infection on in-hospital mortality after excluding patients with hospital stays < 14 days, to reduce the potential for immortal time bias.

All *p* values were two-sided, with *p* < 0.05 considered statistically significant. The data were statistically analyzed using Stata software, version 15.1 (StataCorp, College Station, TX, USA).

### Ethics approval and consent to participate

The study protocol was reviewed and approved by the Research Ethics Committee of the Faculty of Medicine of the Juntendo University (IRB No.19-010). The Research Ethics Committee of the Faculty of Medicine of the Juntendo University waived the need to obtain informed consent from the study participants given the retrospective and anonymized nature of this study in routine care. The JTDB administrators also provided permission to use the data from their database. Our study was performed in accordance with the amended Declaration of Helsinki.

## Results

**Figure 1:**
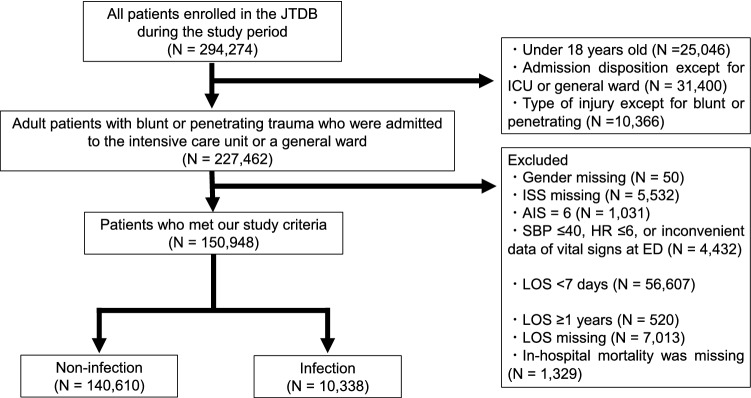
Patient selection. *JTDB* Japan Trauma Data Bank, *ICU* intensive care unit, *ISS* Injury Severity Score, *AIS* Abbreviated Injury Scale, *SBP* systolic blood pressure, *HR* heart rate, *ED* emergency department, *LOS* length of hospital stay.

Of the 294,274 patients in the JTDB from 2004 to 2017, 227,462 adult patients with blunt or penetrating trauma who were admitted to the intensive care unit or a general ward were identified. After eliminating those who met the exclusion criteria, the remaining 150,948 patients were included in this study (Fig. 1).

Of those patients, 10,338 (6.8%) with infection were identified. A total of 1130 (10.9%) patients had sepsis. The demographic characteristics among the patients with and without infection are shown in Table 1. Patients with infection were older than those without [71 (53–82) vs. 67 (47–80) years, *p* < 0.01]. Patients with infection had more comorbidities [6921 (66.9%) vs. 80,450 (57.2%), *p* < 0.01, Supplementary Table 1]. Patients with infection received more emergency procedures [5313 (51.4%) vs. 38,874 (27.6%), *p* < 0.01] and transfusions [3162 (31.0%) vs. 19,077 (13.9%), *p* < 0.01] than those without infection. The use of steroids or immunosuppressants did not differ between the patients with or without infection [48 (0.5%) vs. 532 (0.4%), *p* = 0.17, and 18 (0.2%) vs. 204 (0.1%), *p* = 0.46, respectively]. The severity of trauma was greater in patients with infection than those without [mild, 3901 (37.7%) vs. 84,042 (59.8%); moderate, 4584 (44.3%) vs. 47,743 (34.0%); severe, 1853 (17.9%) vs. 8825 (6.3%), *p* < 0.01].

**Table 1:** Characteristics of trauma patients with and without infection.

	Non-infection	Infection	*p* value
Number	140,610 (93.2)	10,338 (6.8)	
Age	67 (47–80)	71 (53–82)	< 0.01
Gender (male)	81,187 (57.7)	6871 (66.5)	< 0.01
**Mechanism of injury**			
Blunt	136,427 (97.0)	10,070 (97.4)	0.04
Penetrating	4183 (3.0)	268 (2.6)	
**Injury site (AIS ≥ 3)**			
Head	38,793 (27.6)	4082 (39.5)	< 0.01
Face	1008 (0.7)	99 (1.0)	< 0.01
Neck	468 (0.3)	58 (0.6)	< 0.01
Thorax	29,090 (20.7)	2860 (27.7)	< 0.01
Abdomen and pelvis	8184 (5.8)	984 (9.5)	< 0.01
Spine	14,732 (10.5)	1616 (15.6)	< 0.01
Upper extremity	7383 (5.3)	471 (4.6)	< 0.01
Lower extremity	53,510 (38.1)	3940 (38.1)	0.91
Others	35 (0.0)	9 (0.1)	< 0.01
**Trauma severity (ISS category)**			< 0.01
Mild (ISS < 15)	84,042 (59.8)	3901 (37.7)	
Moderate (ISS 15–29)	47,743 (34.0)	4584 (44.3)	
Severe (ISS ≥ 30)	8825 (6.3)	1853 (17.9)	
**Number of comorbidities**			
0	60,160 (42.8)	3417 (33.1)	< 0.01
1	42,810 (30.5)	3197 (30.9)	
2	22,303 (15.9)	1983 (19.2)	
3	9975 (7.1)	1058 (10.2)	
≥ 4	5362 (3.8)	683 (6.6)	
**Medication**			
Steroid	532 (0.4)	48 (0.5)	0.17
Immunosuppressant	204 (0.1)	18 (0.2)	0.46
Anticoagulant	2631 (1.9)	302 (2.9)	< 0.01
**Vital signs at emergency department**			
GCS	15 (14–15)	14 (11–15)	< 0.01
SBP	138 (119–159)	134 (110–158)	< 0.01
HR	82 (71–95)	86 (73–102)	< 0.01
Temperature	36.5 (36.0–37.0)	36.4 (35.8–36.9)	< 0.01
**RR**			< 0.01
≤ 17 (quartile 1)	31,165 (25.9)	1960 (21.2)	
18–23 (quartile 2–3)	54,057 (45.0)	3746 (40.5)	
≥ 24 (quartile 4)	35,042 (29.1)	3537 (38.3)	
**Number of emergency procedures**			
0	101,736 (72.4)	5025 (48.6)	< 0.01
1	26,264 (18.7)	2025 (19.6)	
2	7208 (5.1)	1397 (13.5)	
≥ 3	5402 (3.8)	1891 (18.3)	
**Emergency procedures**			
Intubation	11,837 (8.4)	3257 (31.5)	< 0.01
Ventilator use or assisted ventilation	9461 (6.7)	2476 (24.0)	< 0.01
REBOA	291 (0.2)	105 (1.0)	< 0.01
Chest drainage	8154 (5.8)	1133 (11.0)	< 0.01
Craterization	653 (0.5)	297 (2.9)	< 0.01
Emergency TAE	4050 (2.9)	830 (8.0)	< 0.01
Central venous line use	4262 (3.0)	1454 (14.1)	< 0.01
Vasopressor use	1481 (1.1)	518 (5.0)	< 0.01
Open bone traction	10,228 (7.3)	846 (8.2)	< 0.01
External skeletal fixation	4115 (2.9)	607 (5.8)	< 0.01
Other emergency bone fixation	5430 (3.9)	442 (4.3)	0.04
Blood transfusion	19,077 (13.9)	3162 (31.0)	< 0.01
Any operation	80,186 (57.0)	6883 (66.6)	< 0.01

Continuous variables were compared using the Mann–Whitney U test. Categorical variables were compared using the Chi-square test.

Missing: GCS = 13,085, SBP = 2281, HR = 5577, Temperature = 15,926, RR = 21,441, Blood transfusion = 3421.

*AIS* Abbreviated Injury Scale, *ISS* Injury Severity Score, *COPD* chronic obstructive pulmonary disease, *DM* diabetes mellitus, *HD* hemodialysis, *GCS* Glasgow coma scale, *SBP* systolic blood pressure, *HR* Heart rate, *RR* respiratory rate, *REBOA* resuscitative endovascular balloon occlusion of the aorta, *TAE* transcatheter arterial embolization.

Patients with infection had more concomitant complications than patients without infection (Table 2). Specifically, atelectasis [1048 (10.1%) vs. 906 (0.6%), *p* < 0.01], higher brain dysfunction [954 (9.2%) vs. 2529 (1.8%), *p* < 0.01], and disseminated intravascular coagulation and coagulation disorder [758 (7.3%) vs. 706 (0.5%), *p* < 0.01] were more common in patients with infection than in those without infection.

**Table 2:** Concomitant complications in patients with and without infection.

	Non-infection	Infection	*p* value
Number	140,610 (93.2)	10,338 (6.8)	
**Number of concomitant complications**			
0	129,290 (91.8)	5022 (49.4)	< 0.01
1	9165 (6.5)	2748 (26.6)	
2	1632 (1.2)	1178 (11.4)	
3	360 (0.3)	568 (5.5)	
≥ 4	163 (0.1)	822 (8.0)	
**Central nervous system**			
Diabetes insipidus	194 (0.1)	116 (1.1)	< 0.01
Hydrocephalus	209 (0.2)	166 (1.6)	< 0.01
Fat embolism	84 (0.1)	152 (1.5)	< 0.01
Cerebrospinal fluid leakage	235 (0.2)	115 (1.1)	< 0.01
Higher brain dysfunction	2529 (1.8)	954 (9.2)	< 0.01
Mental disorders (PTSD, etc.)	597 (0.4)	201 (2.0)	< 0.01
Others	1535 (1.1)	512 (5.0)	< 0.01
**Circulation**			
Acute coronary syndrome	63 (0.0)	38 (0.4)	< 0.01
Refractory shock	221 (0.2)	176 (1.7)	< 0.01
Acute kidney injury	186 (0.1)	267 (2.6)	< 0.01
Abdominal compartment syndrome	32 (0.0)	38 (0.4)	< 0.01
Others	759 (0.5)	328 (3.2)	< 0.01
**Respiratory**			
Lung edema	120 (0.1)	167 (1.6)	< 0.01
Atelectasis	906 (0.6)	1048 (10.1)	< 0.01
Pulmonary embolism	289 (0.2)	495 (4.8)	< 0.01
ARDS and respiratory failure	343 (0.2)	618 (6.0)	< 0.01
Others	459 (0.3)	199 (1.9)	< 0.01
**Gastroenterology and hepato-biliary**			
Ulcer and upper GI bleeding	425 (0.3)	325 (3.1)	< 0.01
Ileus	219 (0.2)	159 (1.5)	< 0.01
Pancreatitis	65 (0.1)	52 (0.5)	< 0.01
Hyperbilirubinemia and liver failure	111 (0.1)	165 (1.6)	< 0.01
Others	560 (0.4)	345 (3.3)	< 0.01
**Bone and joint**			
Compartment syndrome	219 (0.2)	346 (3.4)	< 0.01
Refracture	62 (0.0)	342 (3.3)	< 0.01
Pseudoarthrosis	57 (0.0)	377 (3.7)	< 0.01
Others	381 (0.3)	160 (1.6)	< 0.01
**Coagulation**			
DIC and coagulation disorder	706 (0.5)	758 (7.3)	< 0.01
Thrombopenia (< 50,000)	285 (0.2)	347 (3.4)	< 0.01
Others	355 (0.3)	109 (1.1)	< 0.01
**Others**			
Wound disruption	177 (0.1)	321 (3.1)	< 0.01
Decubitus	402 (0.3)	410 (4.0)	< 0.01
Hypothermia (< 35 °C)	203 (0.1)	141 (1.4)	< 0.01
Drug allergy	116 (0.1)	76 (0.7)	< 0.01
Others	1123 (0.8)	428 (4.1)	< 0.01

*PTSD* post trauma stress disorder, *ARDS* acute respiratory destress syndrome, *GI* gastrointestinal, *DIC* disseminated intravascular coagulopathy.

Patients with infection had higher in-hospital mortality [1085 (10.5%) vs. 2898 (2.1%), *p* < 0.01], a longer hospital stay [42 (25–70) vs. 22 (14–38) days, *p* < 0.01], and less discharge at home [2469 (26.7%) vs. 61,702 (44.9%), *p* < 0.01] than patients without infection (Table 3).

**Table 3:** Outcome of trauma patients with and without infection.

	Non-infection	Infection	*p* value
Number	140,610 (93.2)	10,338 (6.8)	
**Admission**			
ICU	79,966 (56.9)	7315 (70.8)	< 0.01
General ward	60,644 (43.1)	3023 (29.2)	
In-hospital mortality	2898 (2.1)	1085 (10.5)	< 0.01
**Place after discharge**			
Home	61,702 (44.9)	2469 (26.7)	< 0.01
Transfer	72,833 (53.0)	6532 (70.7)	
Other	2925 (2.1)	241 (2.6)	
LOS	22 (14–38)	42 (25–70)	< 0.01

Continuous variables were compared using the Mann–Whitney U test. Categorical variables were compared using the Chi-square test.

Missing: place after discharge = 263.

*ICU* intensive care unit, *LOS* length of hospital stay.

**Figure 2:**
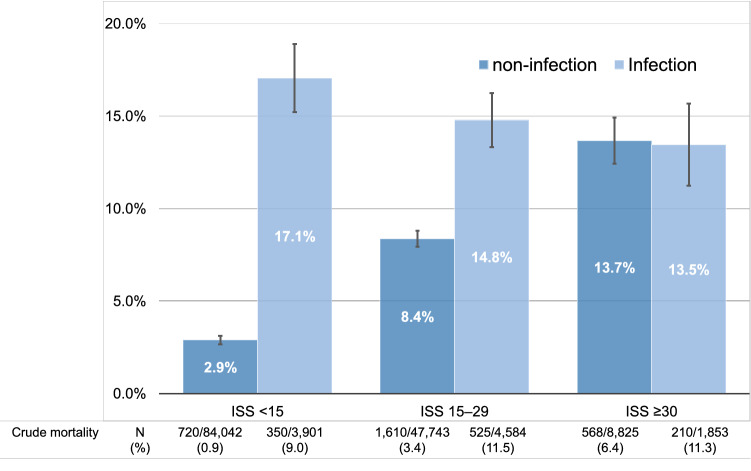
Crude and adjusted in-hospital mortality in patients with and without infection by trauma severity. The crude mortalities are summarized in the table and adjusted mortalities and its 95% confidence interval calculated by the multivariable logistic regression analysis are demonstrated with a bar graph with error bars. *ISS* Injury Severity Score.

Figure 2 shows the effect of infection on trauma patients according to trauma severity. The in-hospital mortality rate between trauma patients with infection and those without infection differed according to trauma severity [17.1% (95% CI 15.2%–18.9%) vs. 2.9% (95% CI 2.7%–3.1%), *p* < 0.01, in patients with mild trauma; 14.8% (95% CI 13.3%–16.3%) vs. 8.4% (95% CI 7.9%–8.8%), *p* < 0.01, in patients with moderate trauma; and 13.5% (95% CI 11.2%–15.7%) vs. 13.7% (95% CI 12.4%–14.9%), *p* = 0.86, in patients with severe trauma]. Details on the multivariable logistic regression analysis are shown in Supplementary Table 2.

We analyzed a subgroup of patients with hospital stays of longer than 14 days from admission to reduce the impact of immortal time bias. Infection occurred in 8.2% of patients. The outcomes in this subgroup analysis were similar to the primary results (Supplementary Table 3). Multivariable logistic regression analysis also showed differences in the impact of infection according to trauma severity: the impact of infections was higher in patients with mild trauma (Supplementary Table 4).

## Discussion

We assessed the impact of infection complications on in-hospital mortality among trauma patients. The impact differed by trauma severity; specifically, infection after trauma increased in-hospital mortality to a greater degree in patients with mild or moderate trauma than in patients with severe trauma.

Among patients with mild or moderate trauma, infection complications were associated with increased in-hospital mortality. Our results were consistent with previous studies using a national database^1^ and a statewide database^16^ in the United States. Although other studies^10,14,19^ did not show the association between infection and in-hospital mortality among trauma, their results have limited generalizability because they were small and single-center studies. Infectious complications in trauma, similar to in postoperative patients^20^ and patients with non-infectious internal diseases^21^, would have a worse impact on their prognosis.

Results of the present study revealed little association between infection complications and increased in-hospital mortality in patients with severe trauma. The results in previous studies^1,16^ were partially inconsistent with our study. They noted that infection complications were associated with increased in-hospital mortality although it was a little effect among patients with severe trauma compared with patients with mild trauma. We believe our study is more accurate because we excluded early trauma death and we adjusted for more important confounders such as transfusion, which were not included in previous studies^1,16^. The lower impact of infection in severely injured patients might have occurred because non-infectious complications had a greater impact on mortality than infectious complications^17^. Infection is a common complication among trauma patients regardless of the injury severity. On the other hand, complications associated with high mortality, including acute respiratory destress syndrome or disseminated intravascular coagulation, rarely occur in mild trauma patients, as shown in Table 2. Infection complication alone might not affect mortality in severely injured patients.

Previous studies^10,17,22^ have reported that the risk for developing infection was high in patients with severe trauma. However, our findings emphasize the importance of paying more attention to the complications of infection, even if the severity of trauma is mild. Patients with less severe disease usually receive less monitoring^23^. Thus, the early recognition of infection plays a key role in managing patients with trauma.

### Limitations

There are some limitations in the present study. First, infection was diagnosed by the physician in charge, which might have resulted in misclassification. Our study included not only septic patients but also patients with non-septic infections. The incidence of infection in patients with trauma in the present study of 6.8%. This incidence may be lower than some previous studies, which included incidences of sepsis from 2 to 15%^1,16,22^. The variety of the study population and study design may have contributed to the different incidence of infection. Second, some complications might have been under-reported, as discussed in our previous study^2^, potentially leading to the overestimation or underestimation of the impact of infection on mortality. Third, we did lacked data on the treatments for both trauma and infection, which might have affected the outcomes. Since 2002, guidelines for trauma care called the Japan Advanced Trauma Evaluation and Care that was created with reference to the Advanced Trauma Life Support^24^ were introduced in Japan. Furthermore, a previous study showed high compliance with the sepsis bundle in Japan^25^. Therefore, we believe that most patients received appropriate treatments. Fourth, immortal time bias may have affected our results because the onset of infection was unknown. To address immortal bias, we excluded patients who died < 7 days from hospital admission. In addition, a subgroup analysis of patients with hospital stays longer than 14 days demonstrated similar findings. Therefore, the effect of immortal bias was not strong enough to change our results. Fifth, a number of concomitant complications were significantly associated with decreased in-hospital mortality (Supplementary Table 2), which might be inconsistent clinically. We could not determine whether the complications had a positive or negative impact on in-hospital mortality because we had no data on the time of the onset of complications. However, we kept these variables in the logistic regression analysis because of their clinical importance. Sixth, infection as the primary cause of death could not be verified because of data limitations. This limitation may have affected our results. Finally, data on the level of consciousness after admission were unavailable. Prolonged disturbance of consciousness might have affected the outcomes.

## Conclusion

Infection complications after trauma affected in-hospital mortality differently according to injury severity. Greater attention to infection complications is necessary among patients with trauma, even if their severity is mild.

## Supplementary Information

Supplementary Tables.

## Data Availability

The datasets during and/or analyzed during the current study available from the corresponding author on reasonable request.
