# Supplementary material for: The impact of infection complications after trauma differs according to trauma severity
Source: Sci Rep. 2021 Jul 5;11:13803. doi: 10.1038/s41598-021-93314-5 (PMC8257796; doi:10.1038/s41598-021-93314-5)
Supplement: Supplementary file 1 — Supplementary Tables. [file 41598_2021_93314_MOESM1_ESM.docx]

Title: The impact of infection complications after trauma differs according to trauma severity

Authors:

Akira Komori, MD, PhD^1, 2,^ Hiroki Iriyama, MD, PhD^1, 2^, Takako Kainoh, MD, PhD^1^, Makoto Aoki, MD, PhD^3, 4^, Toshio Naito, MD, PhD^2^, Toshikazu Abe, MD, MPH, PhD^1, 5^*

Affiliations:

^1^ Department of Emergency and Critical Care Medicine, Tsukuba Memorial Hospital, Tsukuba, Japan

^2^ Department of General Medicine, Juntendo University Faculty of Medicine, Tokyo, Japan

^3^ Advanced Medical Emergency Department and Critical Care Center, Japan Red Cross Maebashi Hospital, Maebashi, Japan

^4^ Department of Emergency Medicine, Gunma University Graduate School of Medicine, Maebashi, Japan

^5^ Department of Health Services Research, Faculty of Medicine, University of Tsukuba, Tsukuba, Japan

Email addresses:

Akira Komori: akg.6412@gmail.com

Hiroki Iriyama: iriyamahiroki@yahoo.co.jp

Takako Kainoh: kitaharatakako@yahoo.co.jp

Makoto Aoki: aokimakoto@gunma-u.ac.jp

Toshio Naito: naito@juntendo.ac.jp

Toshikazu Abe: abetoshi111@gmail.com

Corresponding Author:

Toshikazu Abe, MD, MPH, PhD

Department of Health Services Research, Faculty of Medicine, University of Tsukuba, Tsukuba, Japan

1-1-1 Tennodai, Tsukuba, Ibaraki 305-8577 Japan

Email: abetoshi111@gmail.com

Supplementary Table 1: Type of comorbidities in patients with and without infection

|  | Non-infection | Infection | P-value |
| --- | --- | --- | --- |
| Number | 140,610 (93.2) | 10,338 (6.8) |  |
| Type of comorbidities |  |  |  |
| Ischemic heart diseases | 6,034 (4.3) | 633 (6.1) | <0.01 |
| Heart failure | 3,685 (2.6) | 392 (3.8) | <0.01 |
| Hypertension | 37,922 (27.0) | 3,126 (30.2) | <0.01 |
| Other cardiac diseases | 6,794 (4.8) | 716 (6.9) | <0.01 |
| Asthma | 4,072 (2.9) | 355 (3.4) | <0.01 |
| COPD | 959 (0.7) | 192 (1.9) | <0.01 |
| Other chronic lung diseases | 1,531 (1.1) | 181 (1.8) | <0.01 |
| Liver cirrhosis | 1,010 (0.7) | 138 (1.3) | <0.01 |
| Chronic hepatitis | 2,020 (1.4) | 230 (2.3) | <0.01 |
| Peptic ulcer | 2,980 (2.1) | 283 (2.7) | <0.01 |
| Inflammatory bowel disease | 861 (0.6) | 54 (0.5) | 0.26 |
| Other gastrointestinal diseases | 5,474 (3.9) | 528 (5.1) | <0.01 |
| DM | 16,180 (11.5) | 1,513 (14.6) | <0.01 |
| Obesity | 175 (0.1) | 28 (0.3) | <0.01 |
| Other metabolic diseases | 4,575 (3.3) | 406 (3.9) | <0.01 |
| Stroke | 8,575 (6.1) | 869 (8.4) | <0.01 |
| Psychiatric disease | 8,018 (5.7) | 826 (8.0) | <0.01 |
| Dementia | 9,981 (7.1) | 1,016 (9.8) | <0.01 |
| Other neurological diseases | 3,887 (2.8) | 374 (3.6) | <0.01 |
| AIDS | 28 (0.0) | 1 (0.0) | 0.47 |
| Malignancies | 3,551 (2.5) | 337 (3.3) | <0.01 |
| Hematological diseases | 441 (0.3) | 44 (0.4) | 0.05 |
| HD | 2,283 (1.6) | 244 (2.4) | <0.01 |
| Others | 6,685 (4.8) | 602 (5.8) | <0.01 |

COPD: Chronic obstructive pulmonary disease, DM: Diabetes mellitus, AIDS: Acquired immune deficiency syndrome, HD: Hemodialysis

Supplementary Table 2: Results of multivariable logistic regression analysis in trauma patients (n=147,527)

|  | Odds Ratio | 95% CI | | P-value |
| --- | --- | --- | --- | --- |
| Trauma severity (ISS category) / infection |  |  |  |  |
| Mild (ISS < 15) / absence | Reference | | |  |
| Mild (ISS < 15) / present | 7.09 | 6.13 | 8.21 | <0.01 |
| Moderate (ISS 15–29) / absence | 3.16 | 2.86 | 3.50 | <0.01 |
| Moderate (ISS 15–29) / present | 6.02 | 5.21 | 6.94 | <0.01 |
| Severe (ISS ≥ 30) / absent | 5.50 | 4.79 | 6.30 | <0.01 |
| Severe (ISS ≥ 30) / present | 5.40 | 4.37 | 6.66 | <0.01 |
| Age | 1.04 | 1.03 | 1.04 | <0.01 |
| Sex (male) | 1.33 | 1.23 | 1.43 | <0.01 |
| Number of comorbidities |  |  |  |  |
| 1 | 0.92 | 0.84 | 1.01 | 0.07 |
| 2 | 1.08 | 0.98 | 1.20 | 0.12 |
| 3 | 1.15 | 1.01 | 1.31 | 0.03 |
| >4 | 1.45 | 1.25 | 1.69 | <0.01 |
| Blood transfusion | 1.88 | 1.72 | 2.06 | <0.01 |
| ICU admission | 1.13 | 1.03 | 1.24 | 0.01 |
| Concomitant complications |  |  |  |  |
| Neurological system |  |  |  |  |
| Hydrocephalus | 0.88 | 0.59 | 1.33 | 0.56 |
| Higher brain dysfunction | 0.23 | 0.18 | 0.29 | <0.01 |
| Mental disorders (PTSD, etc.) | 0.33 | 0.19 | 0.58 | <0.01 |
| Fat embolism | 0.82 | 0.38 | 1.77 | 0.62 |
| Cerebrospinal fluid leakage | 0.66 | 0.37 | 1.18 | 0.16 |
| Others | 1.48 | 1.23 | 1.79 | <0.01 |
| Circulation |  |  |  |  |
| Acute coronary syndrome | 12.7 | 7.7 | 20.8 | <0.01 |
| Shock | 0.93 | 0.65 | 1.34 | 0.71 |
| Acute kidney injury | 6.34 | 4.91 | 8.19 | <0.01 |
| Abdominal compartment syndrome | 1.47 | 0.70 | 3.07 | 0.30 |
| Others | 1.88 | 1.49 | 2.38 | <0.01 |
| Respiratory |  |  |  |  |
| Lung edema | 1.50 | 1.03 | 2.18 | 0.04 |
| Atelectasis | 0.63 | 0.51 | 0.79 | <0.01 |
| Pulmonary embolism | 1.64 | 1.11 | 2.43 | 0.01 |
| ARDS and respiratory failure | 3.87 | 3.21 | 4.68 | <0.01 |
| Others | 1.56 | 1.15 | 2.11 | <0.01 |
| Gastroenterology and hepato-biliary |  |  |  |  |
| Ulcer and upper GI bleeding | 1.93 | 1.48 | 2.51 | <0.01 |
| Ileus | 0.87 | 0.55 | 1.36 | 0.53 |
| Pancreatitis | 0.53 | 0.23 | 1.22 | 0.13 |
| Hyperbilirubinemia and liver failure | 2.95 | 2.05 | 4.24 | <0.01 |
| Others | 1.44 | 1.10 | 1.88 | 0.01 |
| Bone and joint |  |  |  |  |
| Compartment syndrome | 0.67 | 0.35 | 1.31 | 0.24 |
| Refracture | 1.46 | 0.43 | 5.03 | 0.55 |
| Pseudoarthrosis | 0.09 | 0.02 | 0.38 | <0.01 |
| Others | 0.81 | 0.45 | 1.48 | 0.50 |
| Coagulation |  |  |  |  |
| DIC and coagulation disorder | 1.94 | 1.59 | 2.35 | <0.01 |
| Thrombopenia (< 50,000) | 1.41 | 1.07 | 1.86 | 0.02 |
| Others | 0.84 | 0.50 | 1.42 | 0.52 |
| Others |  |  |  |  |
| Wound disruption | 0.47 | 0.29 | 0.77 | <0.01 |
| Decubitus | 0.87 | 0.64 | 1.19 | 0.39 |
| Hypothermia (<35 ℃) | 0.67 | 0.42 | 1.06 | 0.09 |
| Drug allergy | 0.64 | 0.30 | 1.37 | 0.25 |
| Others | 0.86 | 0.66 | 1.14 | 0.30 |
| Emergency procedures |  |  |  |  |
| Intubation | 3.53 | 3.16 | 3.94 | <0.01 |
| Ventilation | 1.03 | 0.92 | 1.15 | 0.60 |
| REBOA | 0.82 | 0.55 | 1.22 | 0.32 |
| Chest drainage | 0.51 | 0.45 | 0.58 | <0.01 |
| Thoracentesis | 3.01 | 2.47 | 3.66 | <0.01 |
| Emergency TAE | 0.57 | 0.48 | 0.67 | <0.01 |
| Central venous line use | 0.82 | 0.72 | 0.94 | <0.01 |
| Vasopressor use | 1.95 | 1.66 | 2.28 | <0.01 |
| Open bone traction | 0.88 | 0.75 | 1.04 | 0.14 |
| External skeletal fixation | 0.54 | 0.43 | 0.68 | <0.01 |
| Other emergency bone fixation | 0.55 | 0.44 | 0.70 | <0.01 |
| Operations | 0.59 | 0.54 | 0.63 | <0.01 |

ISS: Injury Severity Score, ICU: Intensive care unit, PTSD: Post trauma stress disorder, ARDS: Acute respiratory destress syndrome, GI: Gastrointestinal, DIC: Disseminated intravascular coagulopathy, REBOA: Resuscitative endovascular balloon occlusion of the aorta, TAE: Transcatheter arterial embolization

Supplementary Table 3: Comparison of outcomes between infected and non-infected patients who had hospital stay over 14 days (n=116,461)

|  | Non-infection | Infection | P-value |
| --- | --- | --- | --- |
| Number | 106,926 (91.8) | 9,535 (8.2) |  |
| Admission |  |  | <0.01 |
| ICU | 59,782 (55.9) | 6,747 (70.8) |  |
| General ward | 47,144 (44.1) | 2,788 (29.2) |  |
| In-hospital mortality | 1,634 (1.5) | 850 (8.9) | <0.01 |
| Place after discharge |  |  | <0.01 |
| Home | 41,838 (39.8) | 2,268 (26.1) |  |
| Transfer | 60,943 (58.0) | 6,202 (71.5) |  |
| Other | 2,300 (2.2) | 206 (2.4) |  |
| LOS | 28 (20–44) | 45 (29–74) | <0.01 |

Continuous variables were compared using the Mann–Whitney U test. Categorical variables were compared using the Chi-square test.

Missing: place after discharge = 220

ICU: Intensive care unit, LOS: Length of hospital stay

Supplementary Table 4: Multivariable logistic regression analysis in trauma patients who had hospital stays over 14 days (n=113,820)

|  | Odds Ratio | 95% CI | | P value |
| --- | --- | --- | --- | --- |
| Trauma severity (ISS category) / infection |  |  |  |  |
| Mild (ISS < 15) / absence | Reference | | |  |
| Mild (ISS < 15) / present | 6.69 | 5.69 | 7.85 | <0.01 |
| Moderate (ISS 15–29) / absence | 2.31 | 2.04 | 2.63 | <0.01 |
| Moderate (ISS 15–29) / present | 5.62 | 4.76 | 6.64 | <0.01 |
| Severe (ISS ≥ 30) / absent | 3.60 | 3.02 | 4.30 | <0.01 |
| Severe (ISS ≥ 30) / present | 5.29 | 4.15 | 6.73 | <0.01 |
| Age | 1.04 | 1.04 | 1.04 | <0.01 |
| Sex (male) | 1.47 | 1.34 | 1.62 | <0.01 |
| Number of comorbidities |  |  |  |  |
| 1 | 0.99 | 0.88 | 1.11 | 0.82 |
| 2 | 1.17 | 1.03 | 1.32 | 0.02 |
| 3 | 1.24 | 1.06 | 1.45 | 0.01 |
| >4 | 1.53 | 1.28 | 1.83 | <0.01 |
| Blood transfusion | 1.91 | 1.70 | 2.13 | <0.01 |
| ICU admission | 1.13 | 1.01 | 1.27 | 0.03 |
| Concomitant complications |  |  |  |  |
| Neurological system |  |  |  |  |
| Hydrocephalus | 0.89 | 0.54 | 1.49 | 0.66 |
| Higher brain dysfunction | 0.28 | 0.21 | 0.38 | <0.01 |
| Mental disorders (PTSD, etc.) | 0.41 | 0.23 | 0.74 | <0.01 |
| Fat embolism | 0.34 | 0.11 | 1.04 | 0.06 |
| Cerebrospinal fluid leakage | 0.65 | 0.31 | 1.39 | 0.27 |
| Others | 1.50 | 1.20 | 1.87 | <0.01 |
| Circulation |  |  |  |  |
| Acute coronary syndrome | 11.0 | 6.30 | 19.1 | <0.01 |
| Shock | 0.78 | 0.50 | 1.21 | 0.26 |
| Acute kidney injury | 6.08 | 4.58 | 8.07 | <0.01 |
| Abdominal compartment syndrome | 1.88 | 0.84 | 4.23 | 0.13 |
| Others | 1.99 | 1.54 | 2.56 | <0.01 |
| Respiratory |  |  |  |  |
| Lung edema | 1.37 | 0.90 | 2.07 | 0.14 |
| Atelectasis | 0.64 | 0.50 | 0.82 | <0.01 |
| Pulmonary embolism | 1.22 | 0.74 | 2.00 | 0.44 |
| ARDS and respiratory failure | 3.43 | 2.76 | 4.26 | <0.01 |
| Others | 1.58 | 1.12 | 2.23 | 0.01 |
| Gastroenterology and hepato-biliary |  |  |  |  |
| Ulcer and upper GI bleeding | 2.25 | 1.70 | 2.98 | <0.01 |
| Ileus | 1.13 | 0.70 | 1.82 | 0.61 |
| Pancreatitis | 0.79 | 0.33 | 1.90 | 0.59 |
| Hyperbilirubinemia and liver failure | 3.52 | 2.40 | 5.17 | <0.01 |
| Others | 1.51 | 1.12 | 2.03 | 0.01 |
| Bone and joint |  |  |  |  |
| Compartment syndrome | 0.71 | 0.33 | 1.52 | 0.38 |
| Refracture | 1.13 | 0.28 | 4.59 | 0.86 |
| Pseudoarthrosis | 0.20 | 0.05 | 0.82 | 0.03 |
| Others | 1.09 | 0.60 | 1.99 | 0.79 |
| Coagulation |  |  |  |  |
| DIC and coagulation disorder | 1.69 | 1.34 | 2.12 | <0.01 |
| Thrombopenia (< 50,000) | 1.57 | 1.15 | 2.16 | 0.01 |
| Others | 0.78 | 0.42 | 1.42 | 0.41 |
| Others |  |  |  |  |
| Wound disruption | 0.57 | 0.35 | 0.94 | 0.03 |
| Decubitus | 1.03 | 0.75 | 1.43 | 0.84 |
| Hypothermia (<35 ℃) | 0.55 | 0.31 | 0.99 | 0.05 |
| Drug allergy | 0.81 | 0.36 | 1.80 | 0.60 |
| Others | 0.89 | 0.66 | 1.21 | 0.47 |
| Emergency procedures |  |  |  |  |
| Intubation | 2.51 | 2.17 | 2.89 | <0.01 |
| Ventilation | 0.91 | 0.79 | 1.05 | 0.21 |
| REBOA | 0.68 | 0.41 | 1.14 | 0.15 |
| Chest drainage | 0.60 | 0.50 | 0.71 | <0.01 |
| Thoracentesis | 1.79 | 1.33 | 2.39 | <0.01 |
| Emergency TAE | 0.64 | 0.53 | 0.79 | <0.01 |
| Central venous line use | 0.91 | 0.77 | 1.08 | 0.30 |
| Vasopressor use | 1.80 | 1.47 | 2.20 | <0.01 |
| Open bone traction | 0.87 | 0.72 | 1.05 | 0.15 |
| External skeletal fixation | 0.65 | 0.50 | 0.84 | <0.01 |
| Other emergency bone fixation | 0.62 | 0.47 | 0.82 | <0.01 |
| Operations | 0.57 | 0.52 | 0.63 | <0.01 |

ISS: Injury Severity Score, ICU: Intensive care unit, PTSD: Post trauma stress disorder, ARDS: Acute respiratory destress syndrome, GI: Gastrointestinal, DIC: Disseminated intravascular coagulopathy, REBOA: Resuscitative endovascular balloon occlusion of the aorta, TAE: Transcatheter arterial embolization
